# Risk factors associated with infection of blood-borne virus among people who used methamphetamine

**DOI:** 10.1186/s12879-020-05464-y

**Published:** 2020-10-09

**Authors:** Yilin Cai, Zheng Dai, Sijin Wen, Ruchi Bhandari

**Affiliations:** 1 Department of Biostatistics, School of Public Health, West Virginia University, One Medical Center Drive, Morgantown, WV 26506 USA; 2 Department of Epidemiology, School of Public Health, West Virginia University, One Medical Center Drive, Morgantown, WV 26506 USA

**Keywords:** Methamphetamine, Blood-borne virus, Polysubstance use, NHANES

## Abstract

**Background:**

The surge of methamphetamine use has been a complicating factor compounding the steeply increasing number of drug overdose deaths in the U.S. Infection from blood-borne viruses including hepatitis B virus (HBV), hepatitis C virus (HCV) and HIV, related to methamphetamine use continue to grow. This study aims to examine the risk factors associated with HBV, HCV and HIV among people who used methamphetamine.

**Methods:**

People who ever used methamphetamine were identified from five National Health and Nutrition Examination Survey (NHANES) cohorts, 2007 to 2016. The outcome was either positive or negative for blood-borne viruses as identified from laboratory tests. Weighted statistics for the combined ten years of data were calculated by multiplying the weighted variable for laboratory measurements by 0.2. We examined the association of sexual activities (sexual partners, sexual identity), drug use behaviors (poly-drug use, injection drug use, frequency of drug use, age started using methamphetamine), demographics, and socio-economic status with blood-borne viruses using bivariate and multivariable logistic regression models.

**Results:**

There were 1132 participants representing approximately 11,996,319 persons who ever used methamphetamine in the U.S. Blood-borne viruses’ positive rate was 13.0 per 100,000. Multivariable logistic regression analyses showed significant associations of blood-borne infections with age 40–49 years (vs. age 20–29 years, adjusted odds ratio 4.77, 95% CI 1.11–20.55), age 50–59 years (vs. age 20–29 years, 10.25, 2.40–43.82), living within poverty index 1–1.9 (vs. poverty index > = 2, 2.55; 1.19–5.49), living below the poverty threshold (vs. poverty index > = 2, 2.55; 1.11–5.86), having lower than high school education (vs. equal or higher than high school education, 3.13; 1.51–6.46), sexual identity as other than heterosexual (vs. heterosexual, 5.60; 1.72–18.28), using methamphetamine and heroin and cocaine (vs. using methamphetamine alone, 4.24; 1.06–16.92), injection drug use (vs. no injection drug use, 3.15; 1.61–6.16), and started using methamphetamine at age above 25 (vs. started using methamphetamine at age between 10 and 17, 2.09; 1.01–4.35).

**Conclusions:**

Among people who use methamphetamine, those who use polysubstance, or who inject substances, are in urgent need for vaccination and interventions to avoid further harm from blood borne infections.

## Background

Methamphetamine-related overdose has been increasing across the United States (U.S.) for the past several years [1]. According to the Centers for Disease Control and Prevention (CDC) data, the overdose death rates of psychostimulants with abuse potential, which primarily include methamphetamine and other drugs such as amphetamine and methylphenidate, had tripled between 2016 and 2017 [2]. Provisional data from CDC indicates that deaths involving psychostimulants continued to increase in 2018, despite a drop in overall overdose deaths observed at the same time [3]. People who use methamphetamine also have elevated risks of nonfatal harms, including mental health disorders [4], violent and aggressive behavior [4], risky sexual behavior [4], sexually transmitted infection [5], harm to a fetus, and infection of blood-borne viruses (BBVs) [4, 6]. The pathogens of primary concern for blood-borne infectious diseases are the human immunodeficiency virus (HIV), hepatitis B virus (HBV), and hepatitis C virus (HCV) [7].

Methamphetamine use has been strongly associated with many outbreaks of blood-borne infections. Of the HBV patients identified in the 2003 HBV outbreak in Natrona County, Wyoming, 88% reported injecting methamphetamine [8]. Methamphetamine use is prevalent among people living with HIV and AIDS, particularly among men who have sex with men in the U.S. [9]. In the 2014 HIV outbreak in Scott County, Indiana, 22% of the patients reported injecting methamphetamine [10]. A study characterizing methamphetamine use and HIV serological status in San Diego found that 54% of the people who used methamphetamine were HIV positive [11]. Another study examined risk factors associated with HBV infections among people who used methamphetamine [12]. A prospective cohort study conducted in Canada determined that injecting methamphetamine predicted HCV infection among young, street-involved persons with injection drug use (IDU) [13].

Other than injection, methamphetamine use through smoking, swallowing, or snorting also increases risk of blood-borne infections by negatively affecting judgment and triggering risky behaviors (e.g., unprotected sex) [14]. In addition to that, it is suggested that long-time methamphetamine use is associated with bleeding gums [15] and increasing risk of blood-borne infections through oral sex among the sexually active population.

Fatal and nonfatal harms caused by rapidly increasing methamphetamine use are further compounded by existing opioid crisis, described by some scholars as “twin epidemics” [16]. Polysubstance use, such as co-occurring use of prescription opioids, synthetic opioids other than methadone, heroin, cocaine, or methamphetamine is now commonplace [1, 17, 18]. Specifically, in 2017, opioids were involved in over half of the 10,333 psychostimulant-related deaths [1]. Deaths involving opioids and methamphetamine significantly increased by 14% between July 2017 and June 2018 [18]. People prefer to use multiple substances for various reasons: (a) to experience the synergistic effect; (b) to enhance the benefits of each substance; (c) to overcome dysphoria and manage withdrawal symptoms; (d) to experiment; (e) to avail cheaper substances; (f) to balance the stimulation from methamphetamine with sedation from opioid/heroin [19, 20]. However, since heroin, fentanyl, and methamphetamine are all short-acting substances, persons with IDU tend to inject more frequently to stay “high”. The combined injection of methamphetamine and opioids, or sequential use of methamphetamine after opioids is associated with an increased number of injections and increased probability of the reuse of syringes, thus, leading to elevated risk of BBVs [21–23].

Under this landscape of increasing polysubstance use, it is not clear how methamphetamine use affects the overall likelihood of blood-borne infections. A dynamic model investigating the excess risk of HIV and HCV infections among people who inject stimulants estimated that a median of 5–10% of new HIV and 3–7% of new HCV infections in the following year could each be attributed to a 10% increase in the prevalence of stimulant injection use [4]. Another recent study found that women, poverty, IDU, and HCV infection were associated with increased risk of HBV infection among people who use methamphetamine [12]. CDC and a few state health departments have developed vulnerability assessment tools to identify counties at high risk of HIV and HCV; however, these tools do not include HBV [21, 24]. To date, no studies have used national data to examine factors associated with overall likelihood of positive BBV test results among people who use methamphetamine.

This study aims to examine risk factors associated with positive BBV test results among people who use methamphetamine in the National Health and Nutrition Examination Survey (NHANES). Findings from this study will identify vulnerable sub-population groups that are susceptible to infections from BBVs.

## Methods

### Study data

The study population was identified from five NHANES cohorts from 2007 to 2016. Conducted by the National Center for Health Statistics (NCHS), NHANES is a continuous cross-sectional survey with data released biannually and is effective in determining the prevalence of major diseases and associated risk factors among adults and children in the U.S. [25, 26]. The NHANES data is rich and unique in two ways. First, it combines information collected from both interviews and physical examinations that are necessary to answer the research questions. The interviews include demographics, socio-economic status, drug use information, and health-related questions; and the physical examinations include medical measurements and results of laboratory tests. Second, each survey cycle examines a nationally representative sample, and findings from the study are generalizable to the U.S. Further details are described elsewhere [26].

### Study population and sampling procedure

**Fig. 1:**
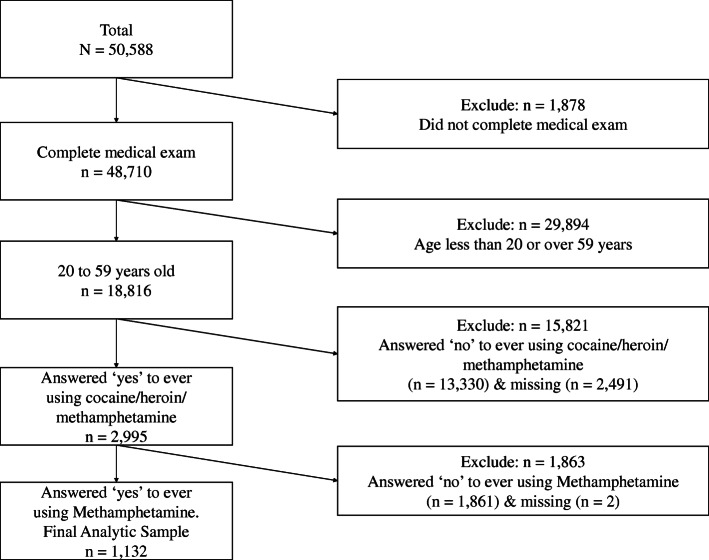
The Flow Chart of Study Case Selection

The study population comprised people who reported methamphetamine use in their lifetime. We chose to use the lifetime methamphetamine use (DUQ330 - ever used methamphetamine) over the most recent methamphetamine use (DUQ350 - last time used methamphetamine) because the latter variable has had up to 95% of data missing. The flow chart in Fig. 1 illustrates the process of case selection. The study included anyone who completed testing for any of the three sets of tests including HBV, HCV and HIV, and also answered “yes” to both questions “ever used cocaine/heroin/methamphetamine” and “ever used methamphetamine”. The study excluded anyone whose age was not between 20 and 59 years as they were not eligible to answer drug use questions.

### Data sources

The primary outcome measure was positive/negative detection of any of the three BBVs (HBV, HCV, and HIV) which were determined according to the results of a set of serological tests. Three HBV serological markers were tested in the NHANES study: antibody to hepatitis B core antigen (anti-HBc), hepatitis B surface antigen (HBsAg), and antibody to hepatitis B surface antigen (HBsAb) [27]. The presence of HBsAg for at least six months indicate a chronic HBV infection [28]. Positive HBV detection was defined as a positive result of anti-HBc; while negative HBV detection was defined as negative for all HBV serological markers including anti-HBc, HBsAg and HBsAb. Indeterminate serological test results were coded as negative since we used a conservative definition to determine positive detection. The HBsAg is tested only when the anti-HBc test is positive. Participants who were HBsAb positive but anti-HBc negative and HBsAg negative were not considered as a population at risk of HBV infection, but susceptible to HIV and HCV.

Two HCV markers were tested: hepatitis C antibody and hepatitis C RNA [29]. The hepatitis C RNA test is only conducted when the hepatitis C antibody test is positive. Current HCV infection was indicated by both hepatitis C antibody and RNA being positive. Chronic HCV infection was defined as hepatitis C RNA positive six months after an acute infection. Positive HCV detection was defined as a positive result for both hepatitis C antibody and hepatitis C RNA, while negative HCV detection was defined as negative for the hepatitis C antibody. Those with a positive antibody HCV test, but a missing RNA test, were also considered negative. Similarly, indeterminate serological test results were coded as negative.

Two HIV serological markers were tested: HIV-1 and HIV-2 antibody [30]. Specimens were initially tested by a combo set of HIV-1/2 Enzyme Immunoassay (EIA), and then repeated reactive specimens are tested with HIV-1/2 supplemental assay. Positive HIV detection was defined as positive results from the two rounds of tests. If EIA is positive but following supplemental tests are not positive (e.g., negative, indeterminate), a confirmatory test is performed for a final decision: HIV detection is positive with a positive confirmatory test result, and HIV detection is negative with a negative confirmatory test result.

According to previous literature [4, 5, 12, 31, 32], demographic characteristics (age, gender, race/ethnicity), socio-economic status (poverty index, health insurance, healthcare access, education), sexual activity (number of sexual partners in the past year, sexual identity), and drug use behaviors (number of drugs used, IDU, number of times methamphetamine used in lifetime, age started using methamphetamine) were known factors associated with infection of BBV. Therefore, these variables were included as potential confounders in the analyses.

Demographics (age, gender, and race), health insurance, hospital utilization, and access to care information were collected through Sample Person Questionnaire. Socio-economic status (poverty index, education) was obtained through Family Questionnaire. Drug use information (e.g., number of drugs used, IDU, number of times methamphetamine used in lifetime, and age started using methamphetamine, etc.) was obtained through Audio Computer Assisted Personal Self Interview (ACASI) Questionnaire. Sexual behaviors (number of sexual partners, sexual identity) were collected through both ACASI and computer assisted personal interview (CAPI) questionnaires during participants’ visit to the examination center. All three BBV-related measures were obtained from corresponding laboratory tests. The specific laboratory methods can be found elsewhere [25]. Responses to questions including education, drug use, and sexual activity were limited to participants aged 20 to 59 years.

### Statistical analysis

Descriptive analyses include both crude and weighted frequency and percentages of all covariates mentioned above. The appropriate sample weights for combined NHANES 2007–2016 data were constructed using National Center for Health Statistics guidelines [33]. Weighted frequencies and percentages were calculated by multiplying the sample weight WTMEC2YR by 0.2. WTMEC2YR is the full sample two year MEC exam weight, which indicates the weighted variable for laboratory measurements. We chose WTMEC2YR as the appropriate weight in our analysis as WTMEC2YR has the least common denominator. The Rao Scott Chi-squared statistic was calculated to assess the association between each covariate and outcome measure. Bivariate logistic regression and three multivariable logistic regression models were developed to examine the risk factors associated with BBV positive results among people who used methamphetamine. The outcome was tested positive for BBV or negative to BBV as identified from laboratory tests. The main risk factors of interest were drug use behaviors (number of drugs used, IDU, number of times methamphetamine used in lifetime, and age started using methamphetamine).

Model I, which only includes demographics, evaluated the effect of demographic characteristics on the BBV positive results. Model II further added a set of socio-economic characteristics and sexual behavior information into the modelling to evaluate their effect on the BBV positive results, controlling for demographics. Although health insurance, healthcare access, and number of sexual partners were not statistically significant in our model, they are, in general, confirmed risk factors for BBV infection according to previous literatures, and they were included in the model to adjust for their effects. Model III further explored how drug use affects the BBV positive results while taking into consideration all previous variables, which is our key research interest. The rationale to include them are two-fold: i), they are statistically significantly associated (*p* < 0.05) with the BBV positive results in the unadjusted analyses; ii), they are suggested to have influence on the likelihood of a positive BBV test.

Unadjusted odds ratios (uORs) and their 95% confidence intervals (CIs) were reported from bivariate logistic regression models, and adjusted odds ratios (aORs) and their 95% CIs were reported from the three multivariable logistic regression models. Missing data were not included in the modelling. For all ORs reported, statistical significance was considered as CI not crossing 1 and corresponding *p*-value being less than 0.05. Chi-square goodness-of-fit test was used to assess the deviance between the statistical models.

**Fig. 2:**
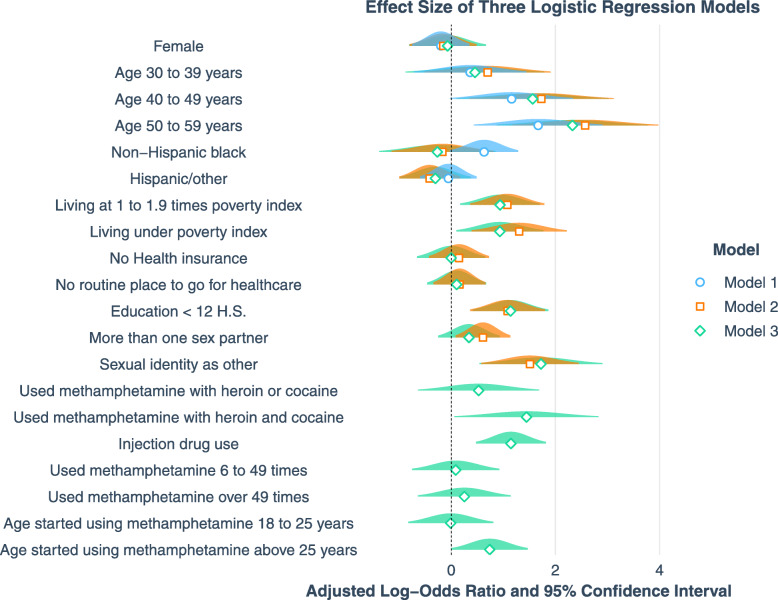
Effect Size of Adjusted Odds Ratios from Three Logistic Regression Models

R programming (RStudio, version 3.6) was used for all analyses. Library “tidyverse” was used to clean data and generate appropriate subsets for statistical analyses. Library “survey” and “srvyr” were used to analyze weighted NHANES data. Survey functions “svytotal”, “svymean”, “svychisq” and “svyCreateTableOne” were used to perform descriptive analyses; “svyglm” was used to perform logistic regression modeling, and “jtools” was used to draw Fig. 2.

## Results

Overall 50,588 people participated in NHANES surveys from 2007 to 2016, of whom 1878 participants who failed to complete medical exams. Further, 29,894 participants who were younger than 20 or over 59 years of age were excluded. There were 18,816 participants eligible to answer the question “ever using cocaine, heroin, or methamphetamine” of whom 2491 (13.2%) participants did not respond to this question. Participants with missing responses were more likely to be female (59% vs. 50%, *p* < 0.001) and belonging to races identified other than white or black (48% vs. 39%, p < 0.001). There were 1132 participants who reported ever using methamphetamine constitute the final analytic sample (Fig. 1), among whom 125 (11%) were BBV positive and 1007 (89%) were BBV negative. Specifically, 213 had HBV vaccine-induced immunity. Among the 125 persons BBV positive, the number of participants infected by HBV, HCV, and HIV were 71 (57%), 78 (62%), and 11 (9%), respectively. Additionally, 35 of them were infected by two viruses: 6 were infected by both HIV and HBV, 29 were infected by both HBV and HCV, and no one was infected by both HCV and HIV. Table 1 summarizes the crude and weighted numbers and percentages of the characteristics of people who used methamphetamine. Based on the weighted estimates, the 1132 participants represented approximately 11,996,319 persons who used methamphetamine in the U.S. population with the overall BBV positive rate at 13.0 per 100,000. Specifically, the positive rate of HBV, HCV and HIV were 6.4, 8.1 and 1.3 per 100,000, respectively.

**Table 1:** Crude and Weighted Numbers and Percentages of the Characteristics of People Who Used Methamphetamine

Variable	Overall		Blood Borne Virus Positive		Blood Borne Virus Negative		***P-value***^***a***^
	N (%)	Weighted frequency (%)	N (%)	Weighted frequency (%)	N (%)	Weighted frequency (%)	
**Number**	1132	11,996,319	125 (11)	1,298,339 (11)	1007 (89)	10,697,980 (89)	
**Gender**							0.457
Female	406 (36)	4,187,779 (35)	43 (34)	398,993 (31)	363 (36)	3,788,786 (35)	
**Age**							**< 0.001**
20–29 years	187 (17)	1,779,073 (15)	4 (3)	68,424 (5)	183 (18)	1,710,649 (16)	
30–39 years	319 (28)	2,968,405 (25)	22 (18)	162,889 (13)	297 (29)	2,805,516 (26)	
40–49 years	330 (29)	3,554,951 (30)	35 (28)	405,851 (31)	295 (29)	3,149,100 (29)	
50–59 years	296 (26)	3,693,889 (31)	64 (51)	661,174 (51)	232 (23)	3,032,716 (28)	
**Race**							0.068
Non-Hispanic White	724 (64)	9,381,391 (78)	81 (65)	1,042,032 (80)	643 (64)	8,339,359 (78)	
Non-Hispanic Black	65 (6)	322,951 (3)	14 (11)	65,729 (5)	51 (5)	257,221 (2)	
Hispanic/Other	343 (30)	2,291,977 (19)	30 (24)	190,577 (15)	313 (31)	2,101,400 (20)	
**Poverty index**							**0.001**
> =2	460 (41)	6,853,711 (57)	26 (21)	454,186 (38)	434 (43)	6,399,526 (62)	
1 to 1.9	325 (29)	2,749,419 (23)	39 (31)	392,263 (33)	286 (28)	2,357,156 (23)	
Below threshold	289 (26)	1,902,201 (16)	50 (40)	351,510 (29)	239 (24)	1,550,691 (15)	
Missing	58 (5)	490,988 (4)	10 (8)	100,380 (8)	48 (5)	390,608 (4)	
**Health insurance**							0.260
No	409 (36)	3,532,006 (29)	50 (40)	451,655 (35)	359 (36)	3,080,350 (29)	
**Healthcare access**							0.353
No	277 (24)	2,606,354 (22)	30 (24)	331,797 (26)	247 (25)	2,274,556 (21)	
**Education**							**0.007**
< 12 High School	259 (23)	2,059,325 (17)	39 (31)	385,837 (30)	220 (22)	1,673,488 (16)	
**Sexual partner**							0.209
< 2	825 (73)	9,005,285 (75)	82 (66)	899,510 (69)	743 (74)	8,105,775 (76)	
> =2	299 (26)	2,947,253 (25)	39 (31)	379,316 (29)	260 (26)	2,567,937 (24)	
Missing	8 (1)	43,782 (0)	4 (3)	19,513 (2)	4 (0)	24,269 (0)	
**Sexual identity**							**0.001**
Heterosexual	1060 (94)	11,287,813 (94)	106 (85)	1,081,527 (83)	954 (95)	10,206,286 (95)	
Other	70 (6)	697,104 (6)	18 (14)	210,072 (16)	52 (5)	487,032 (5)	
Missing	2 (0)	11,402 (0)	1 (1)	6740 (1)	1 (0)	4662 (0)	
**Number of drug use**							**< 0.001**
1 (only methamphetamine)	97 (9)	856,452 (7)	7 (6)	40,539 (3)	90 (9)	815,912 (8)	
2 (methamphetamine + heroin or cocaine)	828 (73)	9,139,692 (76)	57 (46)	728,512 (56)	771 (77)	8,411,180 (79)	
3 (methamphetamine + heroin and cocaine)	207 (18)	2,000,175 (17)	61 (49)	529,288 (41)	146 (14)	1,470,888 (14)	
**Injection drug use**							**< 0.001**
Yes	243 (21)	2,534,473 (21)	80 (64)	701,223 (54)	163 (16)	1,833,250 (17)	
Missing	1 (0)	7107 (0)	1 (1)	7107 (1)	0 (0)	0 (0)	
**Number of times use drug**							**0.014**
< =5 times	352 (31)	3,938,546 (33)	25 (20)	273,084 (21)	327 (32)	3,665,462 (34)	
6–49 times	381 (34)	4,192,770 (35)	36 (29)	406,771 (31)	345 (34)	3,785,998 (35)	
> =50 times	396 (35)	3,848,774 (32)	63 (50)	613,979 (47)	333 (33)	3,234,794 (30)	
Missing	3 (0)	16,230 (0)	1 (1)	4504 (0)	2 (0)	11,725 (0)	
**Age started using methamphetamine**							**0.001**
10–17 years	291 (26)	3,193,550 (27)	33 (26)	319,933 (25)	258 (26)	2,873,617 (27)	
18–25 years	576 (51)	6,382,330 (53)	50 (40)	503,483 (39)	526 (52)	5,878,847 (55)	
> =26 years	261 (23)	2,399,476 (20)	42 (34)	474,923 (37)	219 (22)	1,924,553 (18)	
Missing	4 (0)	20,963 (0)	0 (0)	0 (0)	4 (0)	20,963 (0)	

^a^ *P* value for Rao-Scott χ^2^ test statistic, significance was denoted in bold

In the study sample, slightly over a third (36%) of the participants were female, about two thirds (64%) were non-Hispanic white, 26% of the participants were 50 to 59 years old and they accounted for 51% of BBV positive. Approximately, a quarter (26%) of the participants were living below the poverty threshold and they accounted for 40% of BBV positive cases; another 29% were living between 1 to 1.9 times poverty index and they accounted for 31% of BBV positive cases. Over a third (36%) of the participants did not have any health insurance, and nearly a quarter (24%) did not have routine healthcare access. Nearly a third (31%) of the BBV positive participants had less than high school education. The 6% of participants whose sexual identity was identified as other than heterosexual were accounted for 14% of BBV positive cases. While only 18% of people who used methamphetamine also reported ever using the other two drugs (heroin and cocaine), they accounted for almost a half (49%) of BBV positive cases. The majority of people who used methamphetamine did not inject any drugs (79%); however, almost two thirds (64%) of BBV positive were among the 21% persons with IDU. Over half (51%) of the participants first started using methamphetamine at age 18 to 25, about a quarter (26%) between 10 and 17 years, and another quarter (23%) older than 25 years.

Table 2 summarizes the estimated model effects (uOR and aOR with 95% CIs) of factors associated with the outcome variable. The three multivariable logistic regression models adjusted for the covariates in a stepwise manner. The effect size of all aORs with 95% CIs are illustrated in Fig. 2. In model 1, only being older than 50 (vs. age 20–29, 5.31; 1.54–18.24) was significantly associated with BBV positive cases. After adding socio-economic status and sexual activities into model 2, there were significant associations of BBV positive with age 40–49 years old (vs. age 20–29, 5.64; 1.41–22.62), age 50–59 years old (vs. age 20–29, 13.09; 3.21–53.43), living around poverty index 1 to 1.9 (vs. living 2 times above poverty index, 2.93; 1.44–5.98), living below the poverty threshold (vs. living 2 times above poverty index, 3.68; 1.48–9.17), having lower than high school education (vs. equal to or higher than high school education, 2.95; 1.44–6.05), having more than one sexual partner (vs. having less than two sexual partners, 1.84; 1.09–3.11), and sex identity other than heterosexual (vs. heterosexual, 4.53; 1.78–11.55). In the model 3, after adding drug use behaviors, associations of the same risk factors with BBV positive persisted: age 40–49 years old (vs. age 20–29, 4.77; 1.11–20.55), age 50–59 years old (vs. age 20–29, 10.25; 2.40–43.82), living around poverty index 1 to 1.9 (vs. living 2 times above poverty index, 2.55; 1.19–5.49), living below the poverty threshold (vs. living 2 times above poverty index, 2.55; 1.11–5.86), having lower than high school education (vs. equal to or higher than high school education, 3.13; 1.51–6.46), and sexual identity other than heterosexual (vs. heterosexual, 5.60; 1.72–18.28). In addition, in this model, people who used heroin and cocaine (vs. never used heroin or cocaine, 4.24; 1.06–16.92), IDU (vs. no IDU, 3.15; 1.61–6.16), and started using methamphetamine at ages over 25 (vs. started using methamphetamine between age 10 to 17, 2.09; 1.01–4.35) were also significantly associated with BBV positive. The chi-square goodness-of-fit test suggests that there is no evidence of severe overfitting on the modeling.

**Table 2:** Unadjusted and Adjusted Odds Ratios for Associations between Risk Factors and Blood-borne Viruses’ Positive Results

		Model 1	Model 2	Model 3
Variable	uOR [95% CI]	aOR [95% CI]	aOR [95% CI]	aOR [95% CI]
Gender				
Male^#^	1	1	1	1
Female	0.81 [0.46, 1.41]	0.82 [0.47, 1.42]	0.86 [0.45, 1.63]	0.93 [0.45, 1.95]
Age				
20–29 years^#^	1	1	1	1
30–39 years	1.45 [0.50, 4.20]	1.44 [0.49, 4.23]	2.01 [0.60, 6.75]	1.58 [0.42, 5.99]
40–49 years	3.22 * [1.02, 10.16]	3.19 [0.98, 10.36]	5.64 * [1.41, 22.62]	4.77 * [1.11, 20.55]
50–59 years	5.45 ** [1.63, 18.26]	5.31 * [1.54, 18.24]	13.09 *** [3.21, 53.43]	10.25 ** [2.40, 43.82]
Race				
Non-Hispanic White^#^	1	1	1	1
Non-Hispanic Black	2.05 * [1.06, 3.95]	1.88 [0.98, 3.62]	0.84 [0.32, 2.24]	0.77 [0.25, 2.34]
Hispanic/Other	0.73 [0.43, 1.24]	0.95 [0.55, 1.63]	0.66 [0.37, 1.19]	0.74 [0.37, 1.46]
Poverty index				
> =2^#^	1		1	1
1 to 1.9	2.34 ** [1.29, 4.27]		2.93 ** [1.44, 5.98]	2.55 * [1.19, 5.49]
Below threshold	3.19 ** [1.62, 6.29]		3.68 ** [1.48, 9.17]	2.55 * [1.11, 5.86]
Health Insurance				
Yes^#^	1		1	1
No	1.32 [0.82, 2.13]		1.16 [0.65, 2.07]	1.00 [0.52, 1.92]
Healthcare access				
Yes^#^	1		1	1
No	1.27 [0.77, 2.11]		1.17 [0.71, 1.93]	1.11 [0.63, 1.95]
Education				
> =12 H.S. or GED^#^	1		1	1
< 12 H.S.	2.28 ** [1.27, 4.11]		2.95 ** [1.44, 6.05]	3.13 ** [1.51, 6.46]
Number of sexual partners				
< 2^#^	1		1	1
> =2	1.33 [0.85, 2.07]		1.84 * [1.09, 3.11]	1.41 [0.78, 2.54]
Sexual identity				
Heterosexual^#^	1		1	1
Other	4.07 ** [1.71, 9.68]		4.53 ** [1.78, 11.55]	5.60 ** [1.72, 18.28]
Number of drug use				
1 [only methamphetamine]#	1			1
2 [methamphetamine + heroin or cocaine]	1.74 [0.75, 4.07]			1.69 [0.53, 5.41]
3 [methamphetamine + heroin and cocaine]	7.24 *** [2.93, 17.89]			4.24 * [1.06, 16.92]
Injection drug use				
No^#^	1			1
Yes	5.75 *** [3.40, 9.70]			3.15 ** [1.61, 6.16]
Number of times use drug				
< =5 times^#^	1			1
6–49 times	1.44 [0.71, 2.94]			1.09 [0.47, 2.52]
> =50 times	2.55 ** [1.29, 5.02]			1.29 [0.53, 3.14]
Age started using methamphetamine				
10–17 years^#^	1			1
18–25 years	0.77 [0.39, 1.51]			0.99 [0.44, 2.24]
> =26 years	2.22 ** [1.26, 3.91]			2.09 * [1.01, 4.35]

*Abbreviations*: *uOR* unadjusted odds ratio, *aOR* adjusted odds ratio, *HS* high school, *GED* General Education Development

1. # indicates reference category

2. Statistical significance: *** *p* < 0.001; ** *p* < 0.01; * *p* < 0.05

## Discussion

Findings from our study using 10 consecutive years of NHANES data suggest that polysubstance use and IDU were strongly associated with increased risk of being BBV positive among people who used methamphetamine. Compared with people who were tested BBV negative, those tested BBV positive were largely older, living two times below the poverty index, holding less than a high school education, possessing a sexual identity other than heterosexual, having ever used all three illicit drugs including methamphetamine, heroin, and cocaine, having ever injected drugs, and having started using methamphetamine at ages over 25. Previous literature has shown that people who use methamphetamine have an elevated risk of BBVs infection through sexual risk and injecting risk [9, 34, 35]. Our study found that injection drug use and sexual identity other than heterosexual are significant risk factors associated with elevated risk of BBV infection among this population.

Studies examining polysubstance use and their associations with harmful health effects are usually conducted at a smaller scale and among high-risk populations because of the challenges in capturing such information [19]. Using latent class analysis, studies have illustrated greater occurrences of sexual risk behaviors and increased diagnoses of blood-borne infections [36]. Findings from our study using nationally representative sample with large sample size corroborate results from previous smaller studies that individuals with polysubstance use (i.e., co-ingestion or sequential use of methamphetamine with heroin, fentanyl, or cocaine) have a higher likelihood of being tested BBV positive.

Our study results suggest that persons who started using methamphetamine at ages over 25 were more likely to be tested BBV positive, compared to those who started using methamphetamine at an early age. In a study assessing the effect of age and HIV status on methamphetamine use, the authors conclude that older persons without HIV were using methamphetamine at higher levels and were, therefore, at an increased risk of HIV [11]. More informed knowledge about risky behaviors among vulnerable age-groups can provide guidance to tailor treatment among this subgroup of population.

A recent study concluded that women using methamphetamine were four times more likely to be infected by HBV compared to males [12]. While our study did not find sex statistically significantly associated with BBV positive results, the odds ratio did indicate that women are more likely to be exposed to HBV compared to men. Similarly, the number of past year sexual partners is also likely associated with elevated risk of being tested BBV positive, although not statistically significant.

There are potential limitations associated with this study. Firstly, NHANES participants do not include incarcerated or homeless individuals, who have a higher rate of methamphetamine use than general population. This may affect the generalizability of our conclusion to the entire US adult population. Secondly, for sexual identity, we coded any heterosexual as “heterosexual”, and left all the others as “other than heterosexual”, including men who had sex with men (MSM), female homosexuals, and other sexual identities. Although previous literature has identified MSM as a known risk factor for BBV infection, due to the small sample size of this group (*n* = 26, less than 3%), we categorized MSM into “other than heterosexual”. This recoding method may be different from others and affect the generalizability of our results. Thirdly, questions about illicit drug use and sexual behavior are sensitive in nature, thus, people might refuse to respond or be unwilling to respond honestly to those questions, leading to incorrect estimates. Fourthly, some subgroups have very small sample sizes and therefore, very large confidence intervals. The small sample size potentially limited the power of our study. We had carefully categorized risk factors associated with BBV infection and reduced the effect of the small sample size. Fifthly, we have used “lifetime use of methamphetamine” instead of “most recent use of methamphetamine” as the key case selection variable. The reason is that only 15.8% of the participants in our study reported using methamphetamine in the last year. We recognize that the response to the question of lifetime use of methamphetamine might not be associated with BBV infection temporally. For one, the methamphetamine use could have been many years before any risk for blood-borne infection; for another, the use of methamphetamine could have commenced after the BBV infection.

## Conclusions

In conclusion, as methamphetamine use, especially polysubstance use including methamphetamine, continues to increase, it is of great public health importance to identify those vulnerable populations who are prone to be infected by BBVs. The results of the study are expected to provide evidence to inform timely harm reduction efforts to identify this population and target vaccination and interventions to prevent transmission. Prevention and intervention efforts targeted toward these specific subgroups, including people who use poly-substances, people who inject methamphetamine and/or other substances, people living in low social-economic status, and minorities in sexual identification, can help alleviate fatal and nonfatal harms caused by methamphetamine use. In addition, an evolving polysubstance use landscape indicates a need for a rapid, multifaceted approach to incorporate more comprehensive surveillance efforts to inform effective prevention and response strategies to prevent blood-borne infection outbreaks.

## Data Availability

The datasets generated and/or analysed during the current study are available from R package “nhanesA”, which can be downloaded from: https://cran.r-project.org/web/packages/nhanesA/index.html. The names of the five datasets from 2007 to 2016 are SXQ_I, SXQ_H, SXQ_G, SXQ_F, SXQ_E.

## Abbreviations

AIDS: Acquired Immunodeficiency Syndrome
aOR: Adjusted odds ratio
CDC: Centers for Disease Control and Prevention
CI: Confidence interval
HBsAb: Hepatitis B surface antibody
HBsAg: Hepatitis B surface antigen
HBV: Hepatitis B virus
HCV: Hepatitis C virus
HIV: Human immunodeficiency virus
IDU: Injection drug use
NCHS: National Center for Health Statistics
NHANES: National Health and Nutrition Examination Survey
MSM: Men who had sex with men
uOR: Unadjusted odds ratio
